# Supplementary material for: Antimicrobial Growth Promoters Altered the Function but Not the Structure of Enteric Bacterial Communities in Broiler Chicks ± Microbiota Transplantation
Source: Animals (Basel). 2023 Mar 9;13(6):997. doi: 10.3390/ani13060997 (PMC10044420; doi:10.3390/ani13060997)
Supplement: Supplementary file 1 [file animals-13-00997-s001.zip › animals-2215507_Supplemental.pdf]

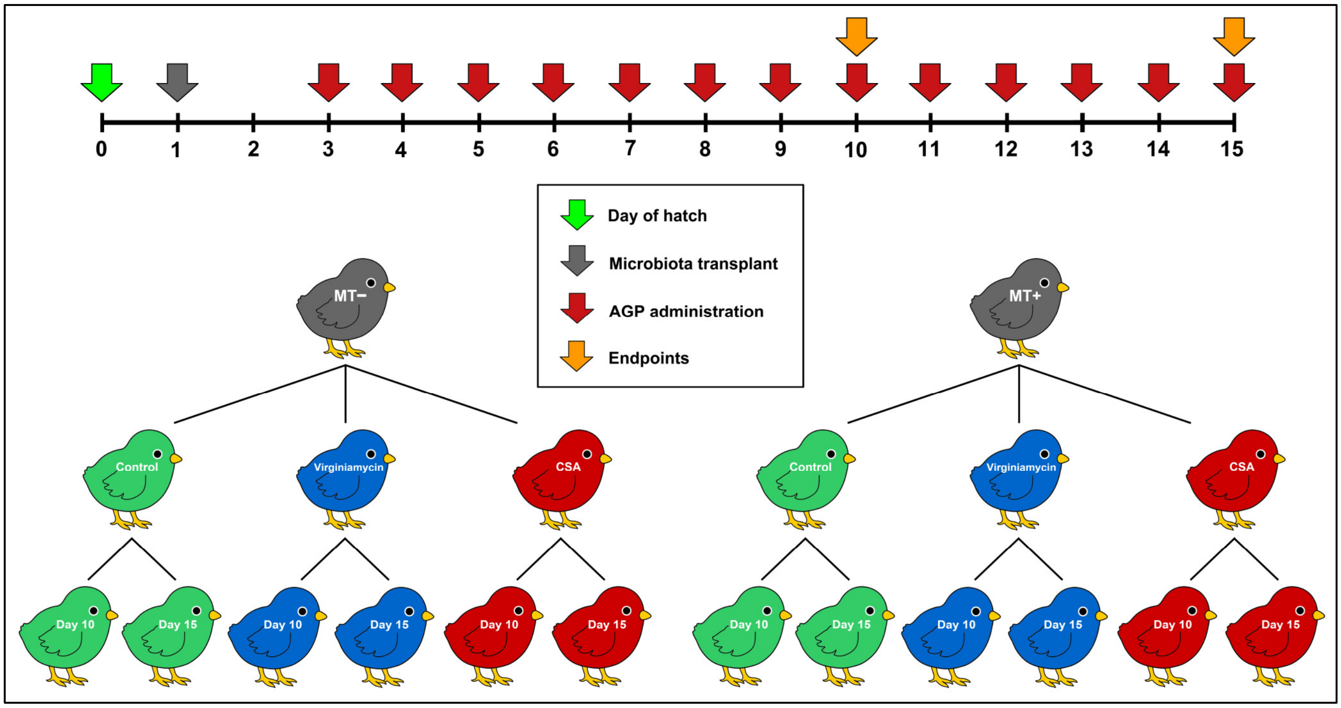

Figure S1. Schematic representation of the experimental design. The experiment was arranged as a two (± microbiota transplant [MT]) by three (antimicrobial growth promoter [AGP], consisting of either the control treatment, virginiamycin, or the ceragenin, CSA-44) by two (endpoint day 10 or day 15) factorial design arranged as a completely randomized design with four replicate chicks per treatment combination (48 birds total). Chicks received a microbiota transplant (MT+) or buffer alone (MT−) at day 1 post-hatch. The AGP treatments were administered continuously throughout the study period commencing when chicks were 3 days-of-age. Birds were maintained in individually ventilated cages. For growth promotion analyses, birds for the two endpoints were combined providing eight replicate chicks per treatment combination (i.e., averaged over the experimental period).

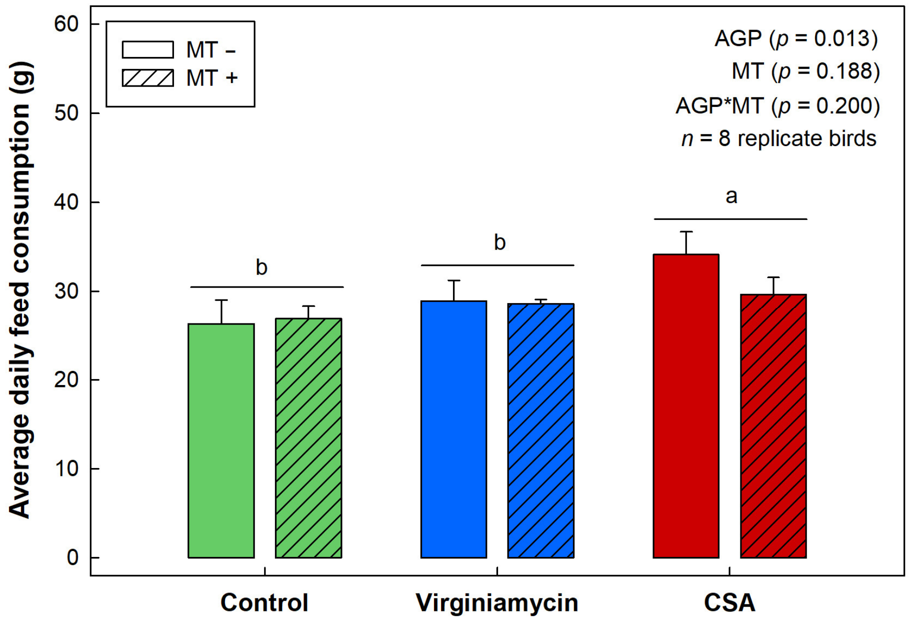

Figure S2. Average daily feed consumption (g) by broiler chicks over the 15 day study period. Chicks were administered an unamended starter diet (Control), a diet supplemented with virginiamycin, or a diet supplemented with the ceragenin, CSA-44 (CSA). Birds received a microbiota transplant (MT+) or buffer alone (MT–) at day 1 post-hatch. Histogram bars not indicated with the same letter differ ($p \leq 0.043$).

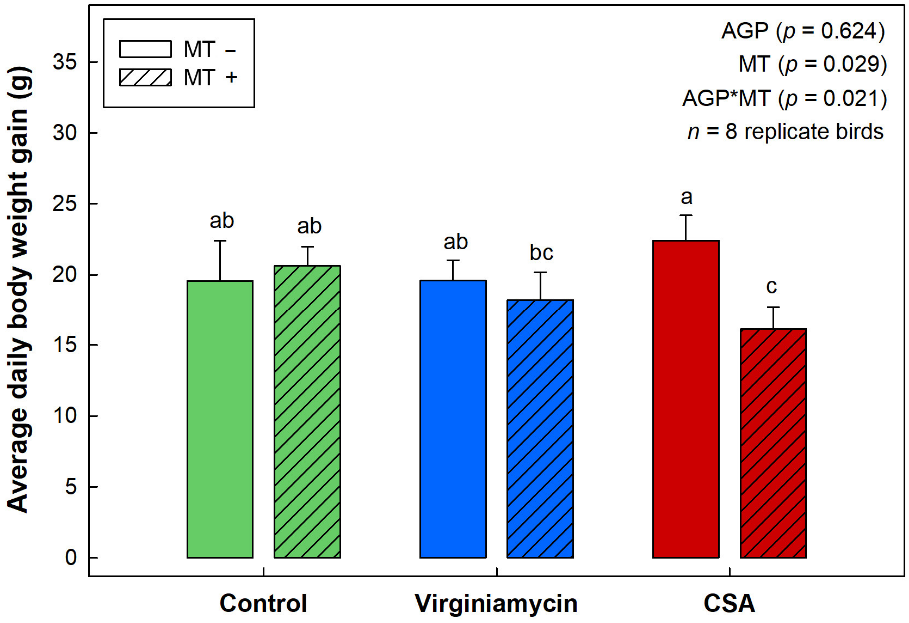

Figure S3. Average daily body weight gain (g) in broiler chicks over the 15 day study period. Chicks were administered an unamended starter diet (Control), a diet supplemented with virginiamycin, or a diet supplemented with the ceragenin, CSA-44 (CSA). Birds received a microbiota transplant (MT+) or buffer alone (MT–) at day 1 post-hatch. Histogram bars not indicated with the same letter differ ($p \leq 0.051$).

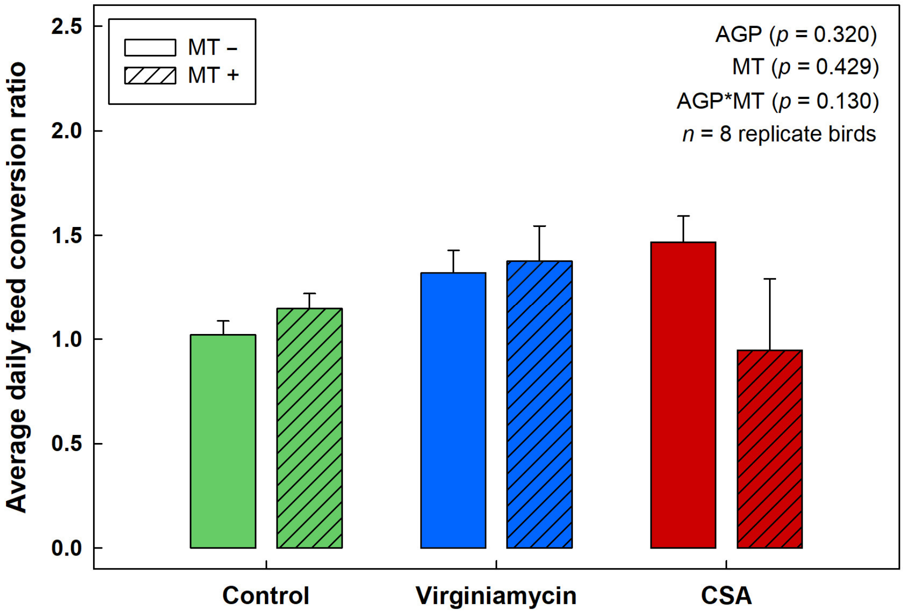

Figure S4. Average daily feed conversion ratio for broiler chicks over the 15 day study period. Chicks were administered an unamended starter diet (Control), a diet supplemented with virginiamycin, or a diet supplemented with the ceragenin, CSA-44 (CSA). Birds received a microbiota transplant (MT+) or buffer alone (MT–) at day 1 post-hatch.

Table S1. Composition of the starter diets used in the study (values in kg).

Ingredient	Control	Virginiamycin	CSA-44
Corn	49.530	49.530	49.530
Soybean meal	43.060	43.060	43.060
Canola oil	2.390	2.390	2.390
Salt	0.510	0.510	0.510
Calcium carbonate	1.520	1.520	1.520
Dicalcium phosphate	1.260	1.260	1.260
Magnesium oxide	0.100	0.100	0.100
L-lysine HCl	0.110	0.110	0.110
D,L-methionine	0.370	0.370	0.370
L-threonine	0.150	0.150	0.150
Vitamin premix	0.100	0.100	0.100
Choline premix	0.100	0.100	0.100
Poultry min premix	0.100	0.100	0.100
Antimicrobial growth promoter[1]	0.000	0.002	0.062

[1]Virginiamycin and the ceragenin, CSA-44 were included at a concentration of 1 and 10 mg/kg of body weight, respectively.
